# Hasselt Corona Impact Study: Impact of COVID-19 on healthcare seeking in a small Dutch town

**DOI:** 10.1038/s41533-025-00426-w

**Published:** 2025-04-06

**Authors:** Carlijn Veldman, Erik A. Van Gijssel, Annelot H. Van Rooij, Lonneke Buitenhuis, Jan Willem K. Van Den Berg, Marco H. Blanker

**Affiliations:** 1 Department of Pulmonary Medicine, Isala Hospital, Zwolle, The Netherlands; 2 Department of Pulmonary Medicine, University Medical Centre Groningen, Groningen, The Netherlands; 3 General practice Van Gijssel, Hasselt, The Netherlands; 4 Community Health Service IJsselland, Zwolle, The Netherlands; 5 Science Academy, Isala Hospital, Zwolle, The Netherlands; 6 Department of Primary and Long-term Care, University of Groningen, University Medical Center, Groningen, The Netherlands

**Keywords:** Health care, Medical research

## Abstract

We investigated healthcare avoidance during the first COVID-19 wave in a Dutch region with high infection rates. A mixed-method, multiphase study used (1) primary care electronic health records to identify patients, (2) questionnaires to capture patients with unreported COVID-19 symptoms, and (3) interviews om care avoidance. Additionally, a natural language model estimated COVID-19 incidence from routine care data. Of 2361 respondents (39% response rate), 535 (23%) reported COVID-19 symptoms; 180 sought help, mainly from GPs. Care-seeking rates did not differ significantly between those with or without relatives who experienced severe illness or death before their own illness (p = 0.270). Interviews showed the main barriers were feeling not ill enough and concerns about an overstressed healthcare system, especially GPs. Only a third of participants with symptoms sought help, mostly from GPs. Serious illness or death of loved ones had no significant impact. Findings highlight the need for clear communication and accessible healthcare, including telemedicine, for future pandemics.

## Introduction

During the first COVID-19 wave, the municipality Hasselt was one of the regions with the highest number of infections in the Netherlands^1^. In response, local general practitioners (GPs) started a contingency plan to prevent overwhelming of the healthcare system^2–4^. Regular and infection related care were separated, and chronic care was paused. Consequently, the number of consultations and diagnoses for other problems, such as cancer or mental illnesses, declined^5–7^. Meanwhile, most appointments were conducted remotely by telephone or video calls unless an in-person appointment was urgently needed^8,9^.

Although some symptoms in primary care are self-limiting, timely medical evaluation is crucial for certain patients to prevent potential complications^10^. The fear of contracting COVID-19, made it more complex to visit a GP. Furthermore, the various restrictive measures could have influenced mental health, possibly leading to changes in healthcare seeking behaviour^6,11,12^.

The underlying reasons for avoiding care during the COVID-19 pandemic remain insufficiently explored. Therefore, the aim of this study was to investigate the impact of COVID-19 on seeking help at the GP during the initial wave of the pandemic, especially for COVID-19 symptoms, in a region heavily affected by the virus. We hypothesized that healthcare-seeking behaviour could be influenced by factors such as the serious illness or death of loved ones. Subsequently, we examined healthcare-seeking behaviour, particularly focused on cases where individuals did not seek help despite having COVID-19 complaints.

## Methods

**Fig. 1:**
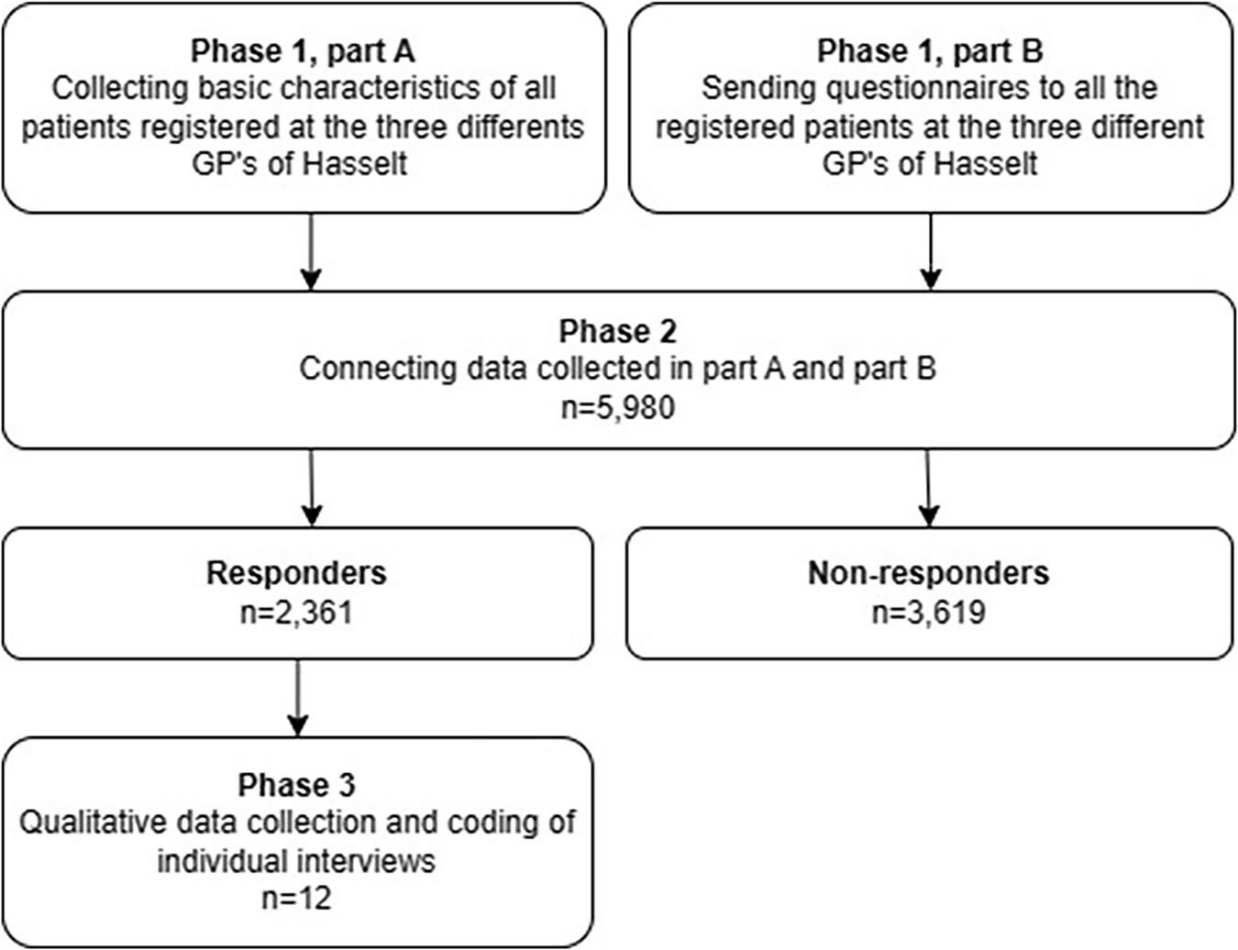
Description of the different phases of our mixed-method study.

We conducted a mixed-method study in a multiphase design (Fig. 1): combining (1) primary care electronic health records to identify patients and collect basic characteristic’s with a (2) questionnaire to identify patients who experienced COVID-19 symptoms but did not seek help and (3) individual semi-structured interviews to uncover the reasons for not seeking help, focusing on the first lockdown period between March 2020 and September 2020. This study was guided by a grounded theory approach.

### Study population

The study population comprised all patients registered at one of the three different general practices in the Dutch municipality Hasselt as of March 2020. They were identified in August 2022, meaning the population had slightly changed since 2020 due to factors such as relocation or mortality. In the Netherlands, all inhabitants are registered with one GP.

### Data collection

Baseline characteristics (March 2020) of all patients were extracted from the electronic health records of the participating practices. Patients with a registered email-address received an online invitation and questionnaire, while others were sent the same materials by post in September 2022 (Supplementary File 1). After two weeks, a reminder was sent to those who received the online questionnaire, but not those who received the postal invitation. Additionally, the study was promoted in the local newspaper “De Stentor”. The questionnaire was designed to identify patients who experienced COVID-19 symptoms during the first wave but did not seek medical care for their COVID-19 complaints. In the absence of previous studies, it was an unvalidated questionnaire developed by several experts in the field.

Due to limited PCR testing available at the beginning of the COVID-19 pandemic, many people were not tested for COVID-19. To estimate the total number of COVID-19 cases within the three general practices, a validated AI model (BERT model), using natural language processing (NLP), was applied^13^. This model was based on the routine care data from the practices, imported in the AHON-registry^14^.

For the interview phase, we applied purposive sampling. Based on survey responses, we identified a subgroup of COVID-19 cases who did not seek medical help. Afterwards, participants from this subgroup were randomly selected and stratified across the three different practices. Subsequently, they were invited to participate in individual, face to face semi-structured interviews at their homes, sometimes with their partner present. An interview guide was developed based on expert opinion and some available literature at that time to explore the participants’ experiences and needs during the first lockdown period. Interviews were conducted by experienced interviewers (AR and ALP) from the regional Community Health Service until no new themes emerged in two consecutive interviews, indicating data saturation. The interviewers were independent of the patients, with no prior relationship or dependency, and had no personal interest in study’s outcomes.

The semi-structured interviews were audio-recorded, transcribed verbatim and analyzed using inductive thematic analysis with Atlas.ti to identify themes^15^. After reading the transcripts, two interviewees (AR and MJ) coded the relevant data for the study. The transcripts were not returned to the participants for review, and no additional interviews were conducted. Related codes were grouped into potential themes derived from the data. Finally, we identified and labeled the themes, and developed a narrative structure accompanied by corresponding descriptions. The interviews and their analysis were conducted in Dutch, and only the results were translated into English, with accuracy through back translation. The original Dutch quotes can be found in Supplementary File 2.

### Statistical analysis

Statistical analysis was performed using SPSS statistics 27.0 software. Characteristics of the study population measured on a continuous scale were represented as either mean ± SD or median (interquartile range), depending on the distribution. Categorical variables were presented as the total number of observations with corresponding percentages. Data comparing participants and non-responders were analyzed by using Mann-Whitney U test or Chi Square tests, depending on the type of data. P-values < 0.05 were considered statistically significant.

### Ethical approval

The study has been approved by the Medical Ethics Committee of the Isala Hospital, Zwolle (registration number 20211017). The study was conducted in accordance with the Declaration of Helsinki. Informed consent was obtained from all participants.

## Results

### Baseline characteristics

In total, 6123 patients aged over 16 years were extracted from three different general practices. 143 patients were excluded due to missing data, leaving 5980 patients in the study population. The response rate of the questionnaire was 39% (n = 2361). Baseline characteristics of participants and non-responders are shown in Table 1. The median age of participants was higher than the non-responders (median 57 years, (range 16–93) vs 40 years (16–93)). Moreover, the proportion of females was higher in the participant group (58%) compared to the non-responder group (44%). Patients with hypertension, heart failure, and malignancies exhibited higher response rates. In contrast, those with asthma or COPD had lower response rates. Lower response rates were observed in individuals identified by the AI model as having COVID-19 symptoms.

**Table 1:** Baseline characteristics responders vs. non-responders.

	Total	Participants	Non-responders
No. of participants	5980	2361 (39.5%)	3619 (60.5%)
**Age** (years, median (IQR))		57 (26%)	40 (31%)
**Sex**			
Female (%)	2978	1374 (58%)	1604 (44%)
Men (%)	3000	985 (42%)	2015 (56%)
Unknown	2	2 ( < 1%)	-
**Registered episodes**^a^			
Diabetes mellitus (%)	357 (5%)	179 (50.1%)	178 (49.9%)
Hypertension (%)	1019 (17%)	571 (56.0%)	448 (44.0%)
Heart failure (%)	74 (1%)	40 (56.3%)	34 (47.7%)
Asthma/COPD (%)	575 (10%)	251 (43.7%)	324 (56.3%)
Malignancy (%)	403 (7%)	234 (58.1%)	169 (41.9)
**GP consultations**^b^			
Consultations GP	4207	1696 (40.3%)	2511 (59.7%)
Medical records with COVID-19	3400	1508 (44.4%)	1892 (55.6%)
Patients with COVID-19	1660	696 (41.9%)	964 (58.1%)

^a^Based on ICPC-codes used in the three different GP’s.

^b^Based on BERT-model^13^.

### COVID-19 symptoms

According to the AI model, out of the 5980 patients, 4207 have had contact with their GP at least once, for various reasons. Information about COVID-19 was discovered in 3400 unique medical records, and out of these, 1508 patients participated in the questionnaire. In 1660 medical records, the AI model predicted the presence of COVID-19. From this group, 41,9% participated in the survey.

Among all participants, 23% (n = 535) reported experiencing any COVID-19 related symptoms (Table 2). Of these, 139 participants (26%) actually had a positive PCR-test by testing at the community health service. The most frequently reported symptoms were cough (56%), fever (56%), dyspnoea (51%), cold (51%) and muscle pain (50%). Among them, 180 participants (33.6%) reported seeking help for COVID-19, with 176 of them contacting their GP. Four patients went directly to the hospital without involving the GP. Of the 535 participants with complaints during the first wave, 236 (44%) reported persistent complaints. Within this group, 80 participants (15%) reported experiencing symptoms for more than 24 months.

**Table 2:** COVID-19 related survey of 39% of all the people in Hasselt, a small Dutch town.

	% of participants (no.)	
**Total no. of participants**	2361	
**Type of living**		
Together with family or other loved ones		86% (n = 2028)
Alone		12% (n = 287)
Nursing home		1% (n = 5)
Unknown		2% (n = 41)
**Highest level of education**		
Primary school		4% (n = 95)
Secondary school (vmbo)		22% (n = 530)
Secondary school (havo)		6% (n = 149)
Secondary school (vwo/gymnasium)		1% (n = 33)
Secondary vocational education (mbo)		33% (n = 782)
Higher vocational education (hbo)		26% (n = 617)
University		5% (n = 121)
Unknown		1% (n = 34)
**Complaints due to COVID-19 (%)**		
No	77% (n = 1824)	
Yes, based on:	23% (n = 535)	
Positive PCR-test		26% (n = 139)
PCR-test was not possible		71% (n = 378)
Didn’t want to do a PCR-test		3% (n = 18)
Not mentioned	<0,1% (n = 2)	
Different COVID-19 related complaints*		
Cough (%)		56% (298/535)
Fever (%)		56% (298/535)
Dyspnoea (%)		51% (275/535)
Cold (%)		51% (275/535)
Muscle pain (%)		50% (270/535)
Sore throat (%)		42% (226/535)
Loss of taste and smell (%)		42% (226/535)
Runny nose (%)		30% (159/535)
Sneezing (%)		18% (95/535)
Nausea (%)		14% (75/535)
Diarrhoea (%)		12% (66/535)
**Participants seeking help due to complaints of COVID-19**		
Not seeking help	66% (355/535)	
Seeking help^**^	34% (180/535)	
GP		31% (166/535)
GP outside office hours		4% (19/535)
Hospital		2% (11/535)
**Treatment for complaints of COVID-19**	14% (75/535)	
Corticosteroids (%)		4% (22/535)
Antibiotics (%)		7% (37/535)
Oxygen suppletion at home (%)		0%
Assessment at the emergency department (without admission) (%)		2% (9/535)
Admission to the hospital (%)		1% (7/535)
**Persistent complaints after COVID-19**	44% (236/535)	
<6 months		19% (100/535)
<12 months		10% (56/535)
<24 months		15% (80/535)

All numbers reflect percentages with participant count.

*Percentages are based on total no. of participants experiencing COVID-19 related symptoms

**The total number is >180: some participants have sought care at more than one place.

Among participants with COVID-19 symptoms, there was no significant difference in the percentage of relatives or acquaintances who experienced severe COVID-19 symptoms or passed away before their own illness, between those who sought care and those who did not (48% vs. 43%, Chi-square p = 0.270). Reasons for not seeking help, as mentioned in the free-text section of the questionnaire, included ‘due to the wave of participants’, ‘we waited at home to reduce the severity of complaints to a bearable level’ and ‘the government’s advice at the time was: only seek for help if you have severe shortness of breath’.

### Thematic analysis semi-structured interviews

**Fig. 2:**
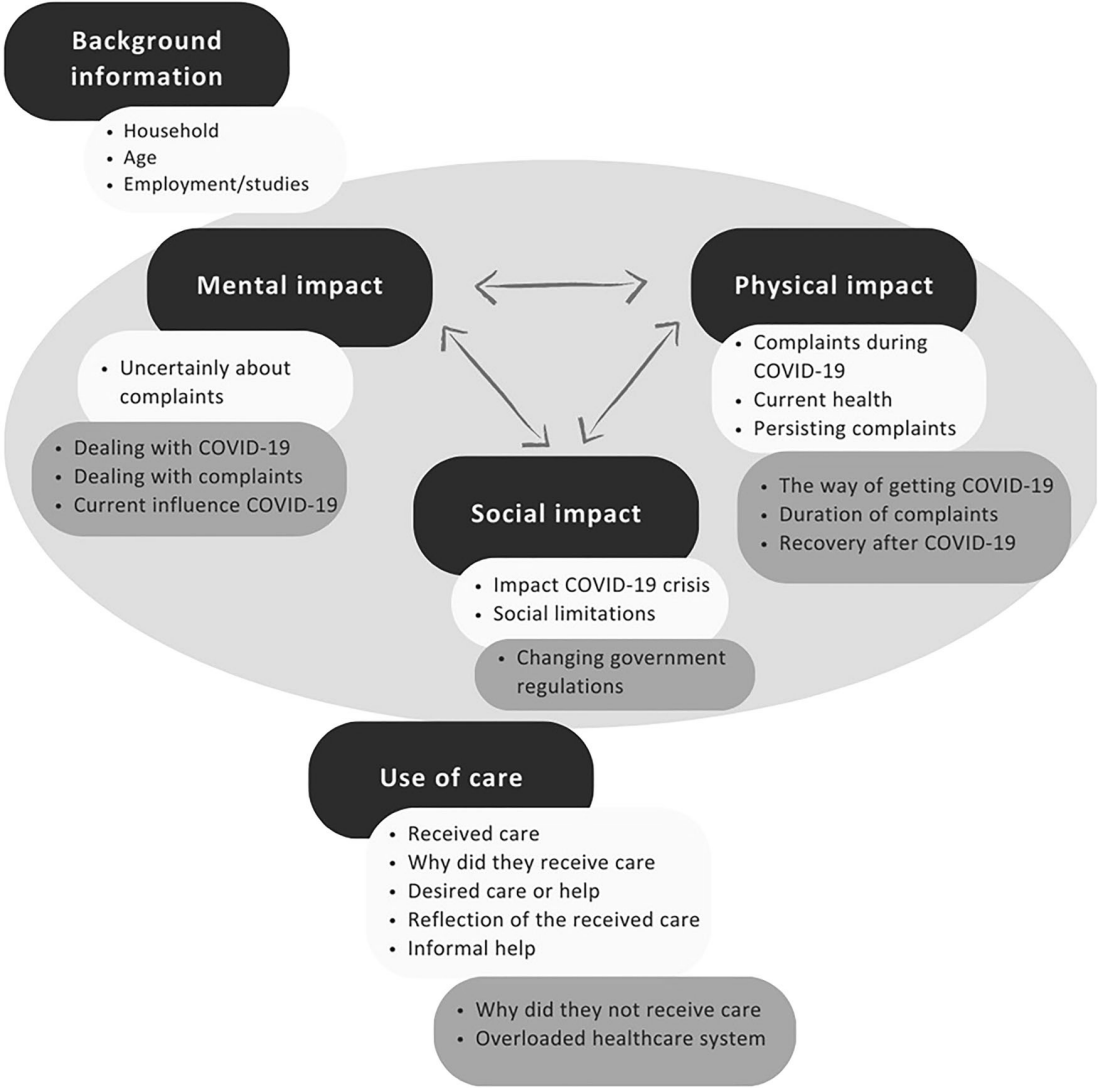
Cross-sectional thematic network of the interrelated themes before the interviews (light grey) and the additional themes resulting from the qualitative analysis (dark grey) of the interviews (n = 12).

Data saturation was reached after interviewing twelve participants. Twenty patients were invited, and twelve were interviewed to reach saturation. The twelve interviewees had a median age of 57 years (range 38–80 years), and generally rated their health as reasonably good (Table 3). Five interviewees (42%) had relatives who experienced severe COVID-19 symptoms or passed away. The average interview duration was twenty minutes, with main themes including mental impact, physical impact, social impact and use of care (Fig. 2).

**Table 3:** Characteristics interviewees.

	Age	Sex	Type of living	Employment	PCR-test	Reached out to GP	Duration severe complaints
**1**.	38	Male	With partner	Working	No	No	One week
**2**.	42	Male	With partner and child(ren)	Working	No	No	A day, less severe three weeks
**3**.	70	Male	With partner and child(ren)	Retired	No	No	Ten days
**4**.	71	Male	With partner	Retired	Yes	Yes (but didn’t receive care from the GP, directly referred to cough clinic)	Five to six weeks
**5**	46	Female	With partner and child(ren)	Working	No	Not during the acute symptoms, but after a few weeks of persisting complaints	Six months
**6**.	59	Male	With partner	Working	No	No	Fourteen days, 9 months tired
**7**.	56	Male	With partner	Working	No	Yes (but didn’t receive care)	Four days, long time with less energy
**8**.	58	Male	With partner and child(ren)	Working	Yes	No	Two weeks
**9**.	38	Female	With partner and child(ren)	Working	No	No	Three days
**10**.	76	Female	With partner	Retired	No	No	Three days, no smell for a year
**11**.	80	Male	Alone	Retired	No	No	Two weeks, less severe two months
**12**.	40	Female	With partner and child(ren)	Working	No	No (only after second infection)	One week, less severe over a year

#### Physical impact

A key reason for not seeking help was participants’ assumption that they were not seriously ill. One participant (#1) said: *I do not go to the GP very often. Actually, to put it differently, I hardly ever go. If I do go, there is a good reason for it, and being sick for three weeks and feeling flu-like does not necessarily mean that I will consult a doctor*.One participant indicated that he was experiencing ongoing complaints but did not want to burden the GP too much due to the high workload for GPs at that time. Participant #5 reflected on the challenging situation for the GPs: *But the knowledge that the GP’s were just in such a crisis and were so terribly busy, and that you indeed first heard one ambulance after another arriving and later one death bell after another, I found that a very bizarre situation, and I do not blame anyone for it, because it was like that*.While most participants were satisfied despite not receiving healthcare, they were able to manage their symptoms on their own. Some indicated they would have sought help if their symptoms had persisted a little longer.

#### Mental impact

The mental impact was mainly attributed to the broader COVID-19 crisis in general. Several participants described the number of deaths on Hasselt as profoundly impactful and intense. Some older participants noted that the infection and crisis had a less impact on them overall, as they were already retired and engaged in fewer activities. Participant #3 stated: *A flu also passes, so I think, this will also pass. And I have a wife who could always step in if necessary, so why would I need extra care?*

#### Social impact

Participants frequently cited social limitations and changes in social interactions as significant factors. A participant (#6) commented on the difficult social limitations as well, but added: *But on the other hand, a huge number of people around you have passed away, everyone has friends and acquaintances, and that had much more impact than me not being able to do my own thing*. Moreover, many participants expressed frustration regarding the lack of clarity from the national government and the frequent changes in COVID-19 regulations, noting that clearer communication could have alleviated these issues. One participant (#4) mentions the lack of appropriate resources to provide good care: *But the fact that it just takes a very long time before they realize that you have corona, while all of Hasselt was infected. If they had just tested everyone back then, it would have been much more accurate. And then you could have taken targeted actions, but now it was just a bit of muddling through*.

#### Use of care

Participants expressed varied preferences for assistance or care. Some desired more support from their GP, but faced barriers like limited complaints and high GP workload. They also highlighted healthcare system bottlenecks worsened by COVID-19, such as limiting PCR testing capacity and insufficient aftercare for long-term COVID-19 effects. Suggestions for improvement included a 24-h helpline, and better guidance on managing complaints and medication use.

Overall, various factors contributed self-direction and self-management among participants. Acceptance of their illness and the inability to visit a GP were common themes. Moreover, the majority experienced spontaneous improvement through rest. One participant (#5) described the situation as*: I am positively surprised by my own ability to deal with it and also to trust the signals my body was giving me and to think, okay, I am sick, I am very sick, but not too sick. I do not need a caregiver at this moment because I am managing*.

Informal support from partners or children, including task assistance and emotional support, helped with self-management. Participants appreciated healthcare workers despite challenges in arranging immediate assistance. While some desired different outcomes, many understood the crisis constraints and did not blame GPs, given the limited understanding of COVID-19 at the time. Looking ahead, participant 5 remarked: *Look, if I had it now, I would just much rather talk directly to a GP, okay, this is what I am noticing now, what is the best course of action, which route should I follow. That bit of consultation, but that simply was not there back then either*.

## Discussion

This mixed-methods study suggests that only one-third of patients who experienced COVID-19 related symptoms during the first wave sought medical care, primarily from their GP. Contrary to our hypothesis, no difference was found between healthcare seeking and the presence of serious illness or death of loved ones. The primary reason for not seeking help was participants’ belief that they were not sick enough, compounded by an overstressed healthcare system, particularly regarding GPs.

The study sample was representative of the Dutch population in terms of comorbidity prevalence^16^. Participants generally expressed contentment, with most managing the situation by themselves. Suggested improvements for future health crises included: sufficient availability of PCR-tests, improved access to health advice (possibly through telephone or digital means), and enhanced information on managing complaints, including long-term symptoms.

The COVID-19 pandemic significantly impacted global mental health, with high rates of anxiety, depression, and stress were reported in the general population^17^. Our study suggests that the mental impact of COVID-19 related symptoms, primarily stemmed from the broader COVID-19 crisis. The uncertainty in this period, reduced healthcare availability healthcare, and limited access to health resources (such as PCR-tests and face masks) influenced participants’ overall well-being. Also, the high number of deaths in this relatively small population has had impact. The relationship between uncertainty and mental health had long been investigated^18^.

Due to the Dutch government-imposed restrictions, access to primary and secondary care was limited, leading to delayed care and missed diagnosis^5–9^. Previous studies have identified several factors influencing care avoidance during the COVID-19 pandemic, including female gender, poor self-perceived health, and high levels of depression and anxiety^2,19^. In our interviews, participants cited social limitations, changes in interactions, and unclear COVID-19 related regulations as key determinants affecting their mental health.

The COVID-19 crisis also prompted an unprecedented restructuring and rapid adaptation of all health and social care givers. For example, healthcare workers increasingly relied on remote care modalities such as telemedicine. Although participants do not want telemedicine to completely supersede face-to-face contacts, participants were satisfied with its use during the pandemic^20^. Furthermore, a UK study among GPs using telemedicine showed that the most commonly cited benefit was time efficiency, which particularly important giving the growing demand for care, especially for GPs^20,21^.

This study provided meaningful insights in healthcare-seeking behavior in a region heavily impacted by infections during the first wave of the COVID-19 pandemic. However, several limitations must be acknowledged. Apart from the interviews, we did not determine how many people would have actually wanted to seek help. Probably some participants who did not seek for help, actually had no desire to seek for help because of few complaints. Second, the severity of the participants’ complaints remain unknown. Furthermore, the questionnaires were sent more than a year after the first wave, which may have caused recall bias. Applying the AI model on the routine care data resulted in a higher number of participants with a probable COVID-19 episode than the participants themselves recalled, meaning the self-reported incidence of COVID-19 could have been underestimated.

## Conclusion

The COVID-19 pandemic has greatly hastened healthcare system transformations. Our study findings can enhance care structures for future pandemics, emphasizing the need for clear communication and accessible healthcare, including telemedicine options.

## Supplementary information

Supplementary File 1

Supplementary File 2

## Data Availability

Data is stored in the online portal ResearchManager. If desired, this can be displayed.
