# Supplementary material for: Hasselt Corona Impact Study: Impact of COVID-19 on healthcare seeking in a small Dutch town
Source: NPJ Prim Care Respir Med. 2025 Apr 6;35:21. doi: 10.1038/s41533-025-00426-w (PMC11972328; doi:10.1038/s41533-025-00426-w)
Supplement: Supplementary file 1 [file 41533_2025_426_MOESM1_ESM.docx]

**Supplementary File 1**

**Let op: deze vragenlijst gaat over de eerste COVID-19 golf die plaatsvond tussen ongeveer januari 2020 en september 2020.**

1. Heeft u in de periode van januari 2020 tot september 2020 klachten gehad die mogelijk kunnen passen bij een besmetting met COVID-19?

- Ja, ik had klachten én een positieve PCR-test
- Ja, ik had wel klachten, maar wilde geen PCR-test doen
- Ja, ik had wel klachten, maar het was niet mogelijk een PCR-test te doen
- Nee ik had geen klachten

1. Welke klachten heeft u gehad die kunnen passen bij een besmetting met COVID-19? U kunt meerder antwoorden aankruisen.

- Hoesten
- Koorts
- Benauwdheid/kortademig
- Verkoudheid
- Loopneus
- Niezen
- Keelpijn
- Verlies van smaak en/of reuk
- Spierpijn
- Misselijkheid
- Diarree
- Overig, namelijk: ……..

1. Heeft u voor uw COVID-19 klachten hulp gevraagd bij de huisarts, huisartsenpost of het ziekenhuis? U kunt meerdere antwoorden aankruisen.

- Ja, namelijk bij de huisarts
- Ja, namelijk bij de huisartsenpost
- Ja, namelijk bij het ziekenhuis
- Nee
- Overig: ….

1. Bent u tussen januari 2020 en september 2020 behandeld voor een (vermoedelijke) COVID-19 infectie? U kunt meerdere antwoorden aankruisen.

- Nee
- Ja, via de huisarts met ontstekingsremmers
- Ja, via de huisarts met antibiotica
- Ja, via de huisarts met zuurstof
- Ja, ik ben ingestuurd naar het ziekenhuis voor behandeling op de Spoedeisende Hulp, maar niet opgenomen
- Ja, ik ben ingestuurd naar het ziekenhuis en opgenomen

1. Kende u in de periode tussen januari 2020 tot september 2020 een naaste met een ernstig beloop van COVID-19?

- Ja, een naaste is overleden aan COVID-19
- Ja, een naaste is opgenomen in het ziekenhuis in verband met COVID-19
- Ja, een naaste is niet opgenomen, maar thuis heel ziek geweest met behandeling via de huisarts
- Nee

1. Wat was de relatie tussen u en uw naaste die besmet was met COVID-19? Kiest het antwoord dat het beste bij de situatie past.

- Gezinslid
- Familielid
- Vriend of vriendin
- Kennis
- Buren
- Anders, namelijk: ……..

1. Was er een naaste ziek voorafgaand aan uw eigen ziekteperiode?

- Ja
- Nee
- Nee, ik ben zelf niet ziek geweest

1. Hoe was uw gezinssamenstelling tussen januari 2020 en september 2020 op het moment van uw COVID-19 klachten?

- Ik woonde alleen
- Ik woonde samen met mijn partner
- Ik woonde samen met mijn partner en kinderen
- Ik woonde samen met een andere familielid dan mijn partner en kinderen
- Ik woonde samen met mijn partner, kinderen en een ander familielid dan mijn partner en kinderen
- Ik woonde samen met iemand anders dan mijn partner of familie
- Ik woonde in een verzorgingstehuis of woongroep

1. Heeft u tijdens de periode tussen januari 2020 en september 2020 gerookt?

- Ja, ik heb tijdens de periode tussen januari 2020 en september 2020 gerookt
- Nee, maar ik heb wel voor de periode tussen januari 2020 en september 2020 gerookt
- Nee, ik heb nooit gerookt

1. Heeft u restklachten ervaren nadat u COVID-19 heeft doorgemaakt?

- Ja, ik heb restklachten ervaren tot 6 maanden (half jaar) na de COVID-19 infectie
- Ja, ik heb restklachten ervaren tot 12 maanden (1 jaar) na de COVID-19 infectie
- Ja, ik heb restklachten ervaren tot 24 maanden (2 jaar) na de COVID-19 infectie
- Nee, ik heb geen restklachten ervaren maar wel besmet geweest met COVID-19 tijdens de periode januari 2020 tot september 2020
- Nee, ik ben niet besmet geweest met COVID-19 tijdens de periode januari 2020 tot september 2020

1. Wat was uw hoogst genoten opleiding tijdens de eerste coronagolf (januari 2020 tot september 2020)?

- Basisschool
- Middelbare school VMBO
- Middelbare school HAVO
- Middelbare school VWO/gymnasium
- Vervolgonderwijs MBO
- Vervolgonderwijs HBO
- Universiteit

1. Bent u bereid om deel te nemen aan een interview gerelateerd aan COVID-19? Indien u kiest voor Ja ontvangt u nadere informatie. U kiest zelf of u mee wilt doen. Nee zeggen kan altijd.

- Ja
- Nee

1. Bent u bereid een tweede vragenlijst in te vullen over uw herstel na een eventuele doorgemaakte COVID-19 infectie?

- Ja
- Nee

Door het invullen van deze vragenlijst gaat u akkoord met het gebruik van uw gegevens voor verder onderzoek naar COVID-19 binnen Hasselt.
