# Supplementary material for: Hasselt Corona Impact Study: Impact of COVID-19 on healthcare seeking in a small Dutch town
Source: NPJ Prim Care Respir Med. 2025 Apr 6;35:21. doi: 10.1038/s41533-025-00426-w (PMC11972328; doi:10.1038/s41533-025-00426-w)
Supplement: Supplementary file 2 [file 41533_2025_426_MOESM2_ESM.docx]

**Supplementary File 2**

***English quote manuscript***

“I do not go to the GP very often. Actually, to put it differently, I hardly ever go. If I do go, there is a good reason for it, and being sick for three weeks and feeling flu-like does not necessarily mean that I will consult a doctor.” (participant 1)

***Original Dutch quote***

“Ik ga niet zo vaak naar de huisarts. Eigenlijk anders gezegd, ik kom er nauwelijks. Als ik er kom, heeft dat een goede reden en drie weken ziek zijn en grieperig voelen, hoeft bij mij nog niet in te houden dat ik een huisarts zal raadplegen.”

***English quote manuscript***

“But the knowledge that the general practitioners were just in such a crisis and were so terribly busy, and that you indeed first heard one ambulance after another arriving and later one death bell after another, I found that a very bizarre situation, and I do not blame anyone for it, because it was like that.” (participant 5)

***Original Dutch quote***

“Maar de wetenschap dat de huisartsen gewoon in zo’n crisis zaten en het zo verschrikkelijk druk hadden en dat je inderdaad eerst de ene ambulance na de andere hoorde aankomen en later de ene doodsklok na de ander, ik vond dat wel een hele bizarre situatie en dat neem ik verder niemand kwalijk, want het was zo”

***English quote manuscript***

*“*A flu also passes, so I think, this will also pass. And I have a wife who could always step in if necessary, so why would I need extra care?” (participant 3)

***Original Dutch quote***

“Een griepje gaat ook voorbij, dus ik denk van, dit gaat ook wel voorbij. En ik heb een vrouw, die altijd als zo nodig wel bij kon springen, dus waarom zou ik nou extra zorg nodig hebben?”

***English quote manuscript***

“But on the other hand, a huge number of people around you have passed away, everyone has friends and acquaintances, and that had much more impact than me not being able to do my own thing." (participant 6)

***Original Dutch quote***

“Maar aan de andere kant, om je heen zijn enorm veel mensen overleden, iedereen heeft vrienden en bekenden, en dat had nog veel meer impact dan dat ik mijn eigen dingetje niet kan doen”.

***English quote manuscript***

"But the fact that it just takes a very long time before they realize that you have corona, while all of Hasselt was infected. If they had just tested everyone back then, it would have been much more accurate. And then you could have taken targeted actions, but now it was just a bit of muddling through." (participant 4)

***Original Dutch quote***

“Maar het feit dat het gewoon heel lang duurt voordat ze doorhebben dat je corona hebt, terwijl heel Hasselt besmet was. Als ze toentertijd iedereen gewoon getest hadden, dan was dat veel accuraat geweest. En dan had je gewoon gericht dingen kunnen doen en nu was het maar een beetje aanmodderen”.

***English quote manuscript***

*“*I am positively surprised by my own ability to deal with it and also to trust the signals my body was giving me and to think, okay, I am sick, I am very sick, but not too sick. I do not need a caregiver at this moment because I am managing.” (participant 5)

***Original Dutch quote***

“Daar ben ik positief verrast door mijn eigen vermogen om daarmee om te gaan en ook te vertrouwen op de signalen die mijn lijf me gaf en te denken oké, ik ben ziek, ik ben heel ziek, maar niet te ziek, ik heb op dit moment geen hulpverlener nodig, want het lukt wel.”

***English quote manuscript***

“Look, if I had it now, I would just much rather talk directly to a GP, okay, this is what I am noticing now, what is the best course of action, which route should I follow. That bit of consultation, but that simply was not there back then either." (participant 5)

***Original Dutch quote***

“Kijk, als ik het nu zou hebben, dan zou ik gewoon veel liever gelijk met een huisarts in gesprek gaan, oké, dit signaleer ik nu allemaal, wat is nu wijsheid, welke route kan ik het beste volgen. Dat stukje overleg, maar dat was er toen ook gewoon niet”.
